# Antithrombotic Management in Patients With Atrial Fibrillation Following Percutaneous Coronary Intervention: An Updated Clinical Review

**DOI:** 10.1002/joa3.70248

**Published:** 2025-12-06

**Authors:** Yuichi Saito, Yoshio Kobayashi

**Affiliations:** ^1^ Department of Cardiovascular Medicine Chiba University Graduate School of Medicine Chiba Japan

**Keywords:** antithrombotic therapy, atrial fibrillation, percutaneous coronary intervention

## Abstract

Patients with atrial fibrillation (AF) often develop acute coronary syndrome and undergo percutaneous coronary intervention (PCI), and vice versa. Acute coronary syndrome and PCI mandate the use of dual antiplatelet therapy, while oral anticoagulation is recommended in patients with AF to mitigate thromboembolic risks. Clinical evidence concerning antithrombotic treatment in patients with either AF or PCI has been accumulated, but when combined, the therapeutic strategy becomes complex. Although triple therapy, a combination of oral anticoagulation with dual antiplatelet therapy, has been employed in patients with AF undergoing PCI as an initial antithrombotic strategy, less intensive regimens may be associated with a lower rate of bleeding without an increased risk of thrombotic events. This narrative review article summarizes currently available evidence of antithrombotic therapy in patients with AF undergoing PCI.

## Graphical abstract

In patients with atrial fibrillation (AF) undergoing percutaneous coronary intervention (PCI), antithrombotic management becomes complex to balance ischemic and bleeding risks. In this context, the recent clinical evidence indicates the “less is more” concept in antiplatelet therapy and oral anticoagulation (OAC). In this review paper, the current understanding and future perspectives in antithrombotic therapy in this setting are summarized.

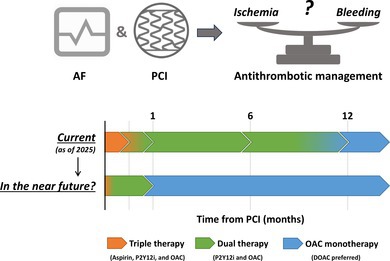

## 1 Introduction

Atrial fibrillation (AF) is a common cardiac rhythm disturbance leading to heart failure and thromboembolic events [1]. The estimated prevalence of AF in Japan and East Asia was approximately 1000 cases per 100,000 population in 2020, projected to rise over the next decades globally [1]. In patients with AF, concomitant coronary artery disease (CAD) is often present, and vice versa. About 20% of patients with AF reportedly develop acute coronary syndrome or undergo percutaneous coronary intervention (PCI) [2]. Although antithrombotic therapies are a cornerstone of the management of patients with AF and those undergoing PCI to prevent thrombotic and ischemic events, the therapeutic regimens become complex when both conditions coexist. Dual antiplatelet therapy (DAPT) is indicated, at least as an initial antiplatelet regimen, in patients undergoing PCI to mitigate the risk of myocardial infarction (MI) and stent thrombosis [3], while oral anticoagulation (OAC) is beneficial in reducing thromboembolic events (e.g., ischemic stroke) to a greater extent than DAPT in patients with AF [4]. Triple antithrombotic therapy, a combination of DAPT plus an OAC, has been employed in clinical practice but is associated with an increased risk of serious bleeding events [5]. According to the results of several randomized controlled trials (RCTs), the guidelines have been updated, showcasing the “less is more” concept [6]. In this context, we provided a narrative review concerning antithrombotic therapy in patients with AF undergoing PCI in 2024 [7], but important RCT data have been reported thereafter. Thus, in the present updated review article, we summarize the current evidence in this literature on antithrombotic therapy in patients with AF undergoing PCI.

## 2 Clinical Trial Results

Since the clinical efficacy of DAPT as compared with OAC was established in RCTs in the late 1990s, DAPT, consisting of aspirin and a P2Y12 inhibitor, has been the cornerstone of antithrombotic management in patients undergoing PCI [3], while OAC with vitamin K antagonist (VKA) was superior to DAPT in reducing ischemic events among patients with AF [4]. In recent decades, direct oral anticoagulation (DOAC) has been shown to have a favorable risk–benefit profile as compared to VKA, particularly in the reduction of intracranial hemorrhage [8]. Thus, a combination of DAPT with OAC may be “theoretically” necessary in patients with AF undergoing PCI. Yet, the clinical effectiveness of this intensive antithrombotic strategy has not been well proven. The term “triple (antithrombotic) therapy” indicates a combination of OAC (VKA or DOAC) plus DAPT (aspirin and a P2Y_12_ inhibitor), while “dual (double) therapy” includes an OAC with single antiplatelet therapy (SAPT) (aspirin or a P2Y_12_ inhibitor) [9]. In addition, OAC monotherapy is a regimen of a single OAC (VKA or DOAC) with no antiplatelet agents.

To date, several RCTs have shown that triple therapy is associated with an increased risk of major bleeding events in patients undergoing PCI with an OAC indication (Table 1) [10, 11, 12, 13, 14, 15, 16, 17]. A meta‐analysis confirmed that DOAC‐based dual (double) therapy after periprocedural triple therapy for 3 to 14 days resulted in a lower risk of major and intracranial hemorrhages as compared with VKA‐based triple therapy for 1 to 12 months, although the less potent antithrombotic regimen (i.e., dual therapy) was probably associated with a higher risk of stent thrombosis [18]. However, dual therapy may be reasonable due to the relatively low event risk of stent thrombosis rather than major bleeding in this patient population. Of note, pivotal RCTs in Table 1, such as the WOEST, PIONEER AF‐PCI, RE‐DUAL PCI, AUGUSTUS, and ENTRUST‐AF PCI trials, randomized patients to either the dual or triple antithrombotic regimen 3 to 14 days after PCI, during which triple therapy was applied even to the experimental dual therapy group. Thus, whether triple therapy, aspirin in particular, can be safely omitted at the time of PCI remains unclear. Another recent meta‐analysis reinforced that periprocedural DAPT was better in terms of bleeding outcomes as compared to short‐term (4–6 weeks) or long‐term (≥ 3 months) DAPT on top of OAC in patients undergoing PCI with an indication for anticoagulation [19].

**TABLE 1:** Key randomized trials of antithrombotic therapy in patients with indications for SAPT and OAC.

	Publication year	Sample size	AF	PCI	Abbreviated DAPT	Prolonged DAPT	Tested OAC	Results
WOEST [10]	2013	573	69%	100%	Periprocedural DAPT followed by clopidogrel	≥ 1 m (BMS) or ≥ 12 m (DES)	VKA	Dual therapy reduced bleeding and death than TT
ISAR‐TRIPLE [11]	2015	614	84%	100%	6 w DAPT followed by aspirin	6 m DAPT (aspirin + clopidogrel)	VKA	6 w TT was not superior to 6 m TT in net clinical outcomes
PIONEER AF‐PCI [12]	2016	2124	100%	100%	Periprocedural DAPT only in the DOAC arm	1–12 m only in the VKA arm	Rivaroxaban vs. VKA^c^	Dual therapy with low‐dose rivaroxaban reduced bleeding events than VKA‐TT
RE‐DUAL PCI [13]	2017	2725	100%	100%	Periprocedural DAPT only in the DOAC arm	1–3 m only in the VKA arm	Dabigatran vs. VKA	Dual therapy with dabigatran reduced bleeding events than VKA‐TT
AUGUSTUS [14]	2019	4614	100%	76%	Periprocedural DAPT followed by P2Y12i	6 m DAPT (aspirin + P2Y12i)	Apixaban vs. VKA	Dual therapy with apixaban reduced bleeding events than VKA‐TT
ENTRUST‐AF PCI [15]	2019	1506	100%	100%	Periprocedural DAPT only in the DOAC arm	1–12 m only in the VKA arm	Edoxaban vs. VKA	Edoxaban‐based dual therapy was noninferior to VKA‐based TT
SAFE‐A [16]	2020	208^a^	100%	100%	1 m DAPT (aspirin + P2Y12i) followed by SAPT	6 m DAPT (aspirin + P2Y12i)	Apixaban	Bleedings were nonsignificantly fewer in abbreviated DAPT group
MASTER‐DAPT [17]	2021	1666^b^	84%	100%	1 m DAPT (aspirin + P2Y12i) followed by SAPT	≥ 3 m DAPT (aspirin + P2Y12i) followed by SAPT	VKA or DOAC	Bleedings were nonsignificantly fewer in abbreviated DAPT group

Abbreviations: AF, atrial fibrillation; DAPT, dual antiplatelet therapy; DOAC, direct oral anticoagulation; OAC, oral anticoagulation; P2Y12i, P2Y_12_ inhibitor; PCI, percutaneous coronary intervention; SAPT, single antiplatelet therapy; TT, triple therapy; VKA, vitamin K antagonist.

^a^ Prematurely terminated due to slow enrollment.

^b^ OAC subanalysis.

^c^ Low‐dose (15 mg daily) and very low‐dose (5 mg daily) rivaroxaban.

Beyond 12 months after PCI, lifelong OAC with no antiplatelet therapy has been recommended in the guidelines according to the historical data with VKA. In this context, RCTs from Japan, the OAC‐ALONE and AFIRE trials, shed light on the evidence gap (Table 2). The OAC‐ALONE was the first RCT that included patients with AF and chronic CAD beyond 1 year after coronary stenting [20]. Although this study was prematurely terminated due to slow enrollment and resulted in insufficient statistical power, a signal of less frequent major bleeding events in the OAC monotherapy group than in the combined OAC plus SAPT group was found [20]. The AFIRE trial demonstrated that rivaroxaban monotherapy was noninferior (and indeed superior) to the combined antithrombotic therapy of rivaroxaban plus SAPT in both ischemic and bleeding endpoints in patients with AF who had undergone PCI with and without drug‐eluting stents (DES) or coronary artery bypass grafting > 1 year earlier or who had angiographically‐confirmed CAD [21]. Although the underlying mechanism of fewer ischemic events in the rivaroxaban monotherapy group in the trial is uncertain, a subanalysis from the AFIRE showed that ischemic outcomes were likely to occur soon after a major bleeding event, which were presumably associated with a decreased threshold for myocardial ischemia and heart failure, potential harm of red blood cell transfusions, and discontinuation of antithrombotic therapy following bleedings [26]. The subsequent EPIC‐CAD trial from Korea showed that edoxaban monotherapy was superior in net adverse clinical events, consisting of all‐cause death, MI, stroke, systemic embolism, unplanned urgent revascularization, or major bleeding or clinically relevant nonmajor bleeding, to the antithrombotic regimen of edoxaban plus SAPT (either aspirin or P2Y12 inhibitor) in patients with AF and CAD, mainly driven by a reduction in bleeding outcomes [22]. In the AFIRE trial, a domestic (reduced) dose of rivaroxaban was employed in Japan, while edoxaban was used at the universally standard dose in the EPIC‐CAD trial. The OAC‐ALONE, AFIRE, and EPIC‐CAD trials were done in an open‐label design and in East Asian patients, where higher bleeding and lower ischemic risk profiles are established as compared to Western populations [27]. Thus, the AQUATIC trial shed further light on this context. This French RCT included patients with chronic CAD who had undergone previous coronary stenting (> 6 months before enrollment) and were receiving long‐term OAC [23]. All participants continued their current OAC and were randomly assigned to receive aspirin or a placebo. The study hypothesis was the superiority of the addition of aspirin on top of OAC in reducing ischemic outcomes. The original target sample size was set at 2000, but the enrollment was terminated early due to an excess of deaths in the aspirin arm. Among 872 patients included in the AQUATIC trial, the primary efficacy outcome, a composite of cardiovascular death, MI, stroke, systemic embolism, coronary revascularization, and acute limb ischemia, occurred in 16.9% and 12.1% in the aspirin and placebo groups during a median follow‐up of 2.2 years (*p* = 0.02). The risk of major bleeding events (10.2% vs. 3.2%, *p* < 0.001) and all‐cause mortality (13.4% vs. 8.4%, *p* = 0.01) were also higher in the aspirin group [23]. The AQUATIC trial reinforced that aspirin should not be added to patients with chronic and stable CAD on top of OAC, but some unresolved clinical questions remain. For example, it is unclear if a P2Y12 inhibitor, rather than aspirin, may convey different safety and efficacy profiles as part of dual therapy, and when antiplatelet therapy can be terminated after PCI. The ADAPT AF‐DES trial from Korea included 960 patients with AF who underwent PCI with DES ≥ 12 months before enrollment and randomized participants to receive DOAC (apixaban or rivaroxaban) monotherapy or dual therapy with DOAC plus clopidogrel. This Korean RCT was characterized by the use of clopidogrel as SAPT and an exclusive patient population treated with contemporary DES. The DOAC monotherapy group was noninferior in net adverse events at 1 year, with superiority in bleeding outcomes, over the DOAC plus clopidogrel group [24]. According to the results of the ADAPT AF‐DES trial, a concomitant antiplatelet agent in addition to an OAC should be stopped at a certain time point irrespective of the drug type (i.e., aspirin or P2Y12 inhibitors) [24]. Another pivotal recent RCT is the OPTIMA‐AF, a multicenter, open‐label RCT in Japan to evaluate the comparable efficacy and safety of short‐term (1‐month DOAC with a P2Y12 inhibitor) and long‐term (12‐month DOAC with a P2Y12 inhibitor) dual therapy followed by DOAC monotherapy in 1079 patients with AF undergoing PCI for chronic CAD or unstable angina [28]. The PCI procedures were performed under intracoronary imaging guidance in almost all cases with cobalt‐chromium everolimus‐eluting stents. This Japanese RCT demonstrated noninferiority in a composite of cardiovascular death, MI, stent thrombosis, stroke, or systemic embolism, and superiority in major or clinically relevant nonmajor bleeding events of the short‐term dual therapy regimen [25]. Although several limitations of the OPTIMA‐AF trial should be acknowledged, including the lower‐than‐expected event rates with a wide noninferiority margin, open‐label design, and exclusion of patients with acute MI, it may be reasonable to omit an antiplatelet agent and to prescribe DOAC alone at 1 month after intracoronary imaging‐guided PCI with DES. From a perspective of cerebrovascular disease, a Japanese RCT also demonstrated that adding SAPT to OAC provided no net clinical benefit over anticoagulant monotherapy, with a higher bleeding risk, in patients with ischemic stroke or transient ischemic attack and concurrent nonvalvular AF and atherosclerotic cardiovascular disease [29]. Probably because of recent advances in the field of cardiovascular medicine [30, 31, 32, 33, 34, 35, 36, 37, 38, 39, 40, 41, 42, 43, 44, 45, 46, 47, 48, 49, 50, 51, 52, 53, 54, 55, 56, 57, 58, 59, 60, 61, 62, 63, 64, 65, 66, 67, 68, 69, 70, 71, 72, 73, 74, 75, 76, 77, 78, 79, 80, 81, 82, 83, 84, 85, 86, 87, 88, 89, 90, 91, 92, 93, 94, 95, 96, 97, 98, 99, 100, 101, 102, 103], ischemic and thrombotic risks after interventional procedures have declined, while bleeding events have increased [104], leading to the “less is more” concept in antithrombotic regimens in the current era.

**TABLE 2:** Key randomized trials of antithrombotic therapy in patients with CAD and indications for OAC.

	Year	Sample size	Country	AF	PCI	Experimental arm	Control arm	Tested OAC	Results
OAC‐ALONE [20]	2018	690^a^	Japan	100%	100%	OAC monotherapy	OAC plus SAPT	VKA or DOAC	Noninferiority of OAC alone strategy was not established
AFIRE [21]	2019	2215	Japan	100%	71%	Rivaroxaban monotherapy	Rivaroxaban plus SAPT	Rivaroxaban	Rivaroxaban monotherapy reduced ischemic and bleeding events
EPIC‐CAD [22]	2024	1040	Korea	100%	60%	Edoxaban monotherapy	Edoxaban plus SAPT	Edoxaban	Edoxaban monotherapy reduced bleeding events
AQUATIC [23]	2025	872^b^	France	89%	100%	OAC plus placebo	OAC plus aspirin	VKA or DOAC	Addition of aspirin versus placebo increased ischemic and bleeding events and mortality
ADAPT AF‐DES [24]	2025	960	Korea	100%	100%	DOAC monotherapy	DOAC plus clopidogrel	DOAC	DOAC monotherapy showed noninferiority in NACE and better outcomes in bleedings
OPTIMA‐AF [25]	2025	1079	Japan	100%	100%	1‐month dual therapy	12‐month dual therapy	DOAC	1‐month regimen was noninferior in ischemic events and superior in bleeding outcomes

Abbreviations: AF, atrial fibrillation; CAD, coronary artery disease; DOAC, direct oral anticoagulation; NACE, net adverse clinical events; OAC, oral anticoagulation; PCI, percutaneous coronary intervention; SAPT, single antiplatelet therapy; VKA, vitamin K antagonist.

^a^ Prematurely terminated due to slow enrollment.

^b^ Terminated early due to an excess of deaths in the aspirin arm.

## 3 Guideline Recommendations and Future Perspectives

**FIGURE 1:**
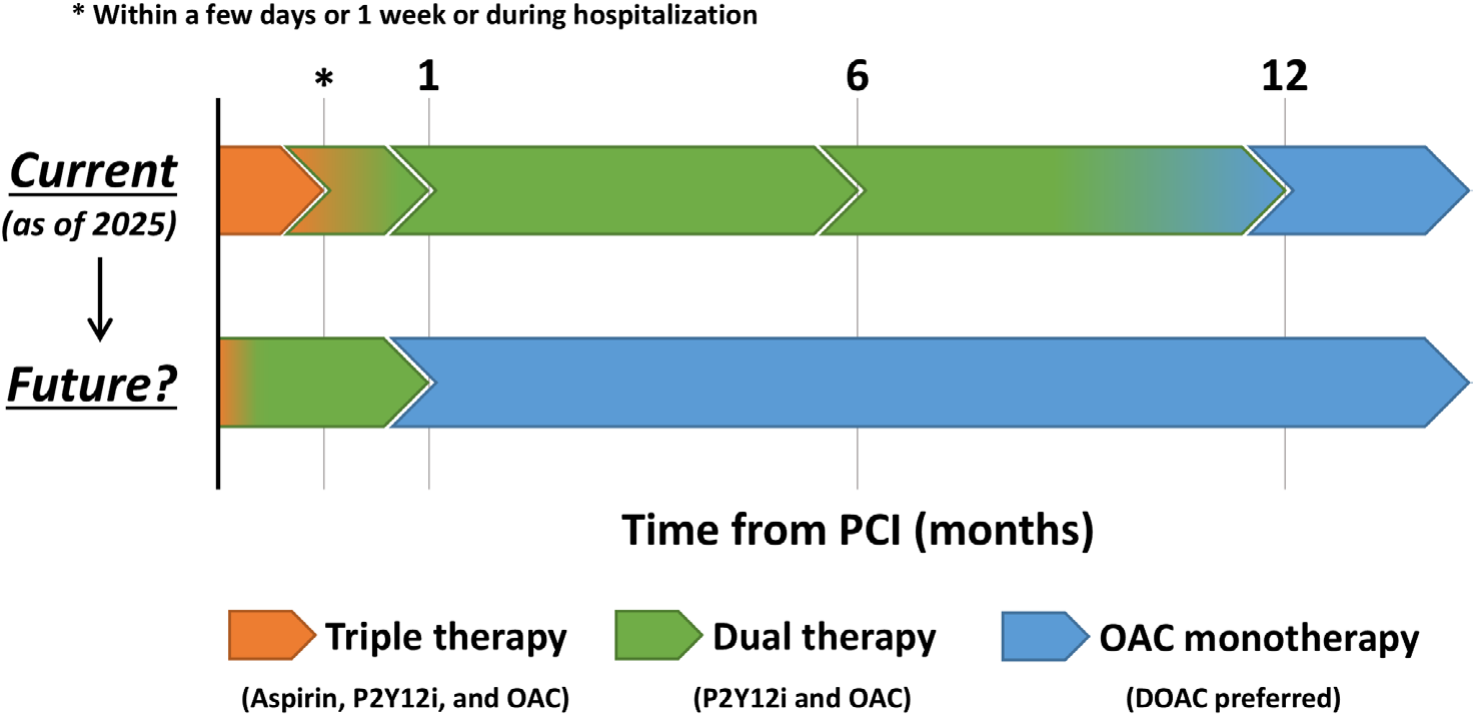
Conceptual algorithm of current and future antithrombotic therapy for patients with atrial fibrillation undergoing PCI. There is considerable overlap among risk factors associated with ischemic and bleeding outcomes, and an antithrombotic regimen should be balanced depending on individual ischemic and bleeding risks. Current guidelines and consensus documents recommend triple therapy (aspirin, a P2Y12 inhibitor, and OAC) for 1 to 30 days after PCI in patients with atrial fibrillation, followed by dual therapy (OAC plus a P2Y12 inhibitor) for (6 to) 12 months and OAC monotherapy thereafter. The potential future antithrombotic regimen includes no or very short‐term DAPT with DOAC, followed by dual therapy with a P2Y12 inhibitor plus DOAC for only 1 month, and DOAC monotherapy thereafter. A P2Y12 inhibitor is preferred as a single antiplatelet therapy. DOAC may be better than a vitamin K antagonist in this setting. DOAC, direct oral anticoagulation; OAC, oral anticoagulation; PCI, percutaneous coronary intervention.

According to the clinical evidence, recent guidelines and consensus documents recommend triple therapy for 1 to 30 days after PCI in patients with AF, followed by dual therapy (OAC plus a P2Y12 inhibitor) for up to 6–12 months and OAC monotherapy thereafter (Figure 1) [9, 105, 106, 107, 108, 109, 110, 111]. Discontinuation of aspirin within 7 days or at discharge is indicated in the recommendations, although triple therapy for up to 30 days is allowed based on individual ischemic and bleeding risks in AF patients undergoing PCI [9]. In terms of intraprocedural anticoagulation in patients undergoing PCI who have an indication for OAC, activated clotting time > 250 s with the use of unfractionated heparin during PCI is recommended [112]. Recent trials have demonstrated that SAPT with a P2Y12 inhibitor rather than aspirin was associated with lower ischemic events after PCI [113, 114, 115], and a P2Y12 inhibitor may be preferable over aspirin as a part of dual therapy with OAC [109]. Additionally, DOAC is recommended over VKA to reduce intracranial hemorrhage [9, 109]. Lifelong OAC monotherapy is indicated since 6–12 months after PCI in the current guidelines (Figure 1). Of note, accurate ischemic and bleeding risk stratification is relevant in clinical decision‐making in antithrombotic therapy in patients undergoing PCI with AF, although no risk‐scoring systems have been established in this context. From a PCI perspective, the criteria of the Academic Research Consortium for High Bleeding Risk (ARC‐HBR) are a guideline‐recommended tool and are used globally for bleeding risk stratification, while the Japanese guidelines recommend domestically modified criteria of ARC‐HBR in patients undergoing PCI [109]. However, whether antithrombotic management guided by the ARC‐HBR criteria improves clinical outcomes in patients undergoing PCI remains unclear. At present, ischemic and bleeding risk stratification using such risk scores may be reasonable, but further studies are warranted.

For the future direction, the authors believe that the “less is more” concept will advance further. Although recent trials have investigated “aspirin‐free” regimens from the beginning of PCI in patients with stable and unstable CAD in a setting of no OAC indications and led to mixed results [116, 117, 118, 119], aspirin as a part of triple therapy may be safely omitted, at least in a setting of low thrombotic risk. Ongoing study results, including the LEGACY (NCT05125276) and PREMIUM (NCT05709626) trials, will reshape our understanding of the role of antiplatelet therapy in PCI [120]. In patients with AF undergoing PCI, several RCTs, such as the MATRIX‐2 (NCT05955365) and WOEST‐3 (NCT04436978) trials, are currently underway to evaluate the safety and efficacy of different antithrombotic regimens [120, 121]. The MATRIX‐2 trial has been done in European countries and Brazil, which is similarly designed to the OPTIMA‐AF trial, while the WOEST‐3 trial uniquely evaluates the clinical relevance of temporary OAC omission within 30 days after PCI. Even though the current guidelines recommend a combination of SAPT with OAC for 6–12 months after PCI, a regimen of no or 1‐day DAPT with DOAC, followed by dual therapy with a P2Y12 inhibitor plus DOAC for 1 month, and DOAC monotherapy thereafter may be possible (Figure 1). Furthermore, left atrial appendage occlusion and resection will potentially play a significant role in this field, although the current evidence is not abundant to support the prioritized use of the technologies and techniques [122, 123].

## 4 Conclusions

Although the antithrombotic regimen should be balanced depending on ischemic and bleeding risks, enthusiastic efforts on antithrombotic therapy in recent decades have established the “less is more” concept in AF patients undergoing PCI. Future investigations will provide better guidance for antithrombotic regimens and standards for this patient population.

## Funding

The authors have nothing to report.

## Disclosure

Yuichi Saito has received lecture fees from Daiichi Sankyo and Novartis. Yoshio Kobayashi has received lecture fees from Amgen, Novartis, Medtronic Japan, and Daiichi Sankyo, and research grants from Abbott Medical Japan, Win International, Nipro, Kaneka Medics, and OrbusNeich Medical.

## Conflicts of Interest

The authors declare no conflicts of interest.
